# 

**Published:** 1898-07

**Authors:** 


### CONTENTS.

COMMUNICATIONS.

"Orthodontia" ... 301
The Mouth a Human Culture Tube ... 306
Irregularities of the Dental Arch—Abstract ... 310
Presidential Address—Abstract ... 316
Temporo-Maxillary Articulation.—Abstract ... 322
National Dental Association, Southern Branch ... 335
Resolutions Adopted by the New York Institute of Stomatology at a Meeting Held on May 3rd, 1898 ... 336

SELECTIONS.

The Camera in Astronomy ... 309
Inflammation of the Sinuses of the Face ... 321
Ptomaines ... 333
Are We All Fools? ... 334

COMMENCEMENTS.

Cincinnati College of Dental Surgery ... 337
Detroit College of Medicine ... 337
Marion-Sims College of Medicine, Dental Department ... 338
Ohio College of Dental Surgery ... 338
University of Michigan, Dental Department ... 339
Northwestern University Dental School ... 340
University of Pennsylvania Dental Department ... 341
Commencement University of Minnesota ... 342
Kansas City Dental College ... 343
List of Committee for the Joint Meeting of the Southern Branch of the National Dental Association and the Louisiana State Dental Society ... 343

EDITORIAL.

Dentists in the Army ... 344
Meetings ... 346
Appointment—Dental Board ... 347
American Medical Association—Section on Stomatology ... 347
Tri-State Meeting ... 348
National Dental Association Meeting ... 349
An Important Request ... 349
The National Association of Dental Faculties ... 350
The National Dental Association ... 350

